# Dynamics of perinatal bovine leukemia virus infection

**DOI:** 10.1186/1746-6148-10-82

**Published:** 2014-04-04

**Authors:** Gerónimo Gutiérrez, Irene Alvarez, Ramiro Merlini, Flavia Rondelli, Karina Trono

**Affiliations:** 1 Instituto de Virología, Centro de Investigaciones en Ciencias Veterinarias y Agronómicas, INTA, CC 1712, Castelar, Argentina; 2 Cátedra de Inmunología, Facultad de Ciencias Veterinarias, Universidad Nacional de Rosario, Rosario, Argentina

**Keywords:** BLV, Perinatal infection, Proviral load, Natural infection

## Abstract

**Background:**

Bovine leukemia virus (BLV) is highly endemic in many countries, including Argentina. As prevention of the spread from infected animals is of primary importance in breaking the cycle of BLV transmission, it is important to know the pathophysiology of BLV infection in young animals, as they are the main source of animal movement. In this work, we determined the proviral load and antibody titers of infected newborn calves from birth to first parturition (36 months).

**Results:**

All calves under study were born to infected dams with high proviral load (PVL) in blood and high antibody titers and detectable provirus in the colostrum. The PVL for five out of seven calves was low at birth. All animals reached PVLs of more than 1% infected peripheral blood mononuclear cells (PBMCs), three at 3 months, one at 6 months, and one at 12 months. High PVLs persisted until the end of the study, and, in two animals, exceeded one BLV copy per cell. Two other calves maintained a high PVL from birth until the end of the study. Antibody titers were 32 or higher in the first sample from six out of seven calves. These decayed at 3–6 months to 16 or lower, and then increased again after this point.

**Conclusions:**

Calves infected during the first week of life could play an active role in early propagation of BLV to susceptible animals, since their PVL raised up during the first 12 months and persist as high for years. Early elimination could help to prevent transmission to young susceptible animals and to their own offspring. To our knowledge, this is the first study of the kinetics of BLV proviral load and antibody titers in newborn infected calves.

## Background

Bovine leukemia virus (BLV) is highly endemic in many countries, including Argentina [1], where important economic losses result from fatal lymphosarcoma and restricted trade of genetic material. Because of the high prevalence, classical control measures, such as test and elimination and/or test and segregation, are not economically sustainable. Therefore, infection transmission should be decreased as much as practicable, to eventually reduce prevalence and drive the infection to extinction. A feasible alternative, previously discussed [1], could be achieved by eliminating animals with high proviral loads (PVLs), most of them with persistent lymphocytosis (PL) [2,3], and as consequence, with a higher probability of transmission of infection [4], when compared to aleukemic (AL) animals. As prevention of spread from infected animals is of primary importance in breaking the cycle of BLV transmission, it is important to know the pathophysiology of BLV infection in young animals, as they are the main source of animal movement. In this work, we followed the PVL and antibody (Ab) titers of infected newborn calves from birth to first parturition, to better understand BLV infection evolution before calves enter the milking herd, when cattle are commingled and infection is likely to spread.

## Results

**Figure 1:**
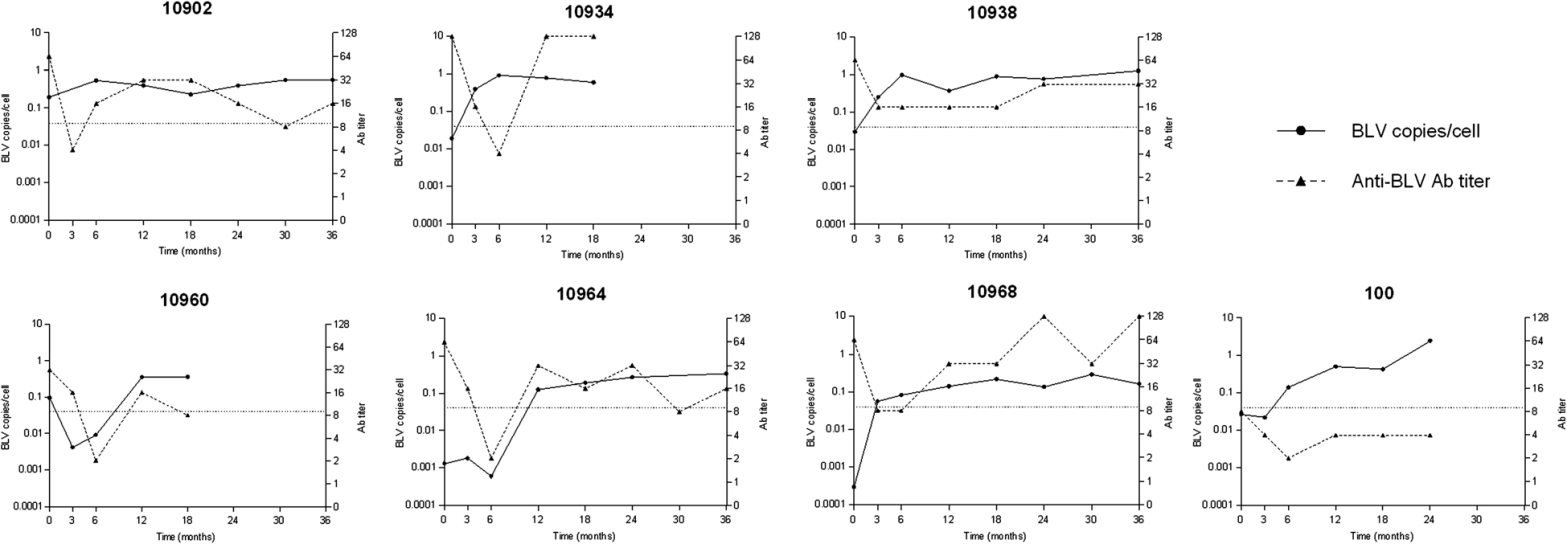
**Kinetics of proviral load and antibody titers in new born infected calves.** A cut-off value of 0.04 BLV copies/cell, corresponding to 1% BLV-infected PBMCs, is the threshold between low and high PVL according to our own criteria and published data.

**Figure 2:**
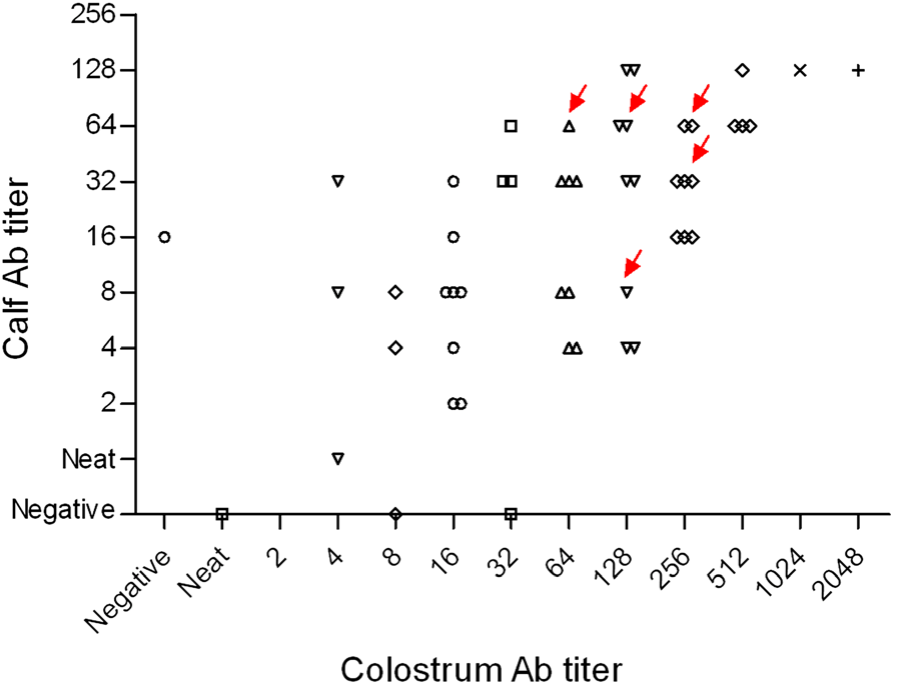
**Antibody titers in maternal colostrum and calves plasma.** Scatter plot showing the distribution of anti-BLV antibody titers in 51 colostrum-calf paired samples belonging to the dairy farm where all animals (except for calf #100) were born. Maternal colostrum and calves antibody titers were significantly different (Mann Whitney, *P* < 0.0001) and showed a positive correlation (Spearman rank test, r = 0.638). Arrows indicate values of the calves under study.

Seven newborn infected calves were followed (Figure 1). The PVLs of five out of seven calves were low at birth. All animals reached PVL levels of more than 1% infected peripheral blood mononuclear cells (PBMCs) (Figure 1); three calves at 3 months, one calf at 6 months, and one calf at 12 months. High PVLs persisted until the end of the study (Figure 1). Calf #10902 maintained a high PVL from birth until the end of the study (36 months) and calf #10960 showed PVL fluctuations with a sporadic decrease under the PVL threshold of 0.04 BLV copies/cell at 3–6 months of age. The PVL of calves #10938 and #100 rose to more than 1 BLV copy/cell. Antibody titers were 32 or higher in the first sample from all calves except for calf #100 that showed the lowest antibody titer at birth (8). Antibody titers decreased at 3–6 months to 16 or lower and then rose for the remainder of the study. Six calves (#10902, #10934, #10938, #10960, #10964 and #10968) belonged to a dairy farm with a high BLV prevalence; these represent 11% of the calves born in the herd during 2 months. The dams of these calves had high PVL in blood (6/6) (Table 1) and high antibody titers (Figure 2) and detectable provirus (4/5) in the colostrum (Table 1). Antibody levels detected in the calves’ blood confirmed good colostrum intake and antibody absorption corresponding to highest antibody titers in the group (Figure 2). Three animals could not be followed over the 36 months; animal #10934 died at 18 months from unrelated causes, #10960 was sold, and #100 was 2 years old at the conclusion of this study.

**Table 1:** Proviral load, antibody titers, and nested PCR results from maternal blood and colostrum

		**Blood**	**Colostrum**
**ID dam**	**Id calf**	**BLV copies/cell**	**Nested PCR**
1203	10964	0.4761	+
1278	10902	ND	+
9442	10938	0.9392	-
10226	10968	0.3164	ND
26744	10934	0.4237	ND
26900	10960	0.1499	+
535	100	1.0870	+

ND: not determined, sample not available, +: positive, -: negative.

## Discussion

We followed the dynamics of BLV infection in calves that acquired infection during the first week of life. While the study size is limited, our data show that perinatal infection can rapidly lead to PVLs similar to highly infected adult cattle, and more than one copy of BLV provirus per cell (Figure 1). Whether this is due to infection dose, animal age, or the mechanism of transmission remains unclear but suggests that newborn calves could become optimal BLV propagators to neighboring cattle. While we cannot confirm the infection source, the decay of antibodies at 3–6 months (Figure 1) suggests that infection was not acquired *in utero* but by contact during delivery or via consumption of infected colostrum or milk. This is supported by the fact that all dams had high blood PVLs and the majority had detectable provirus in colostrum (Table 1), even in the presence of high antibody titers (Figure 2). This finding is consonant with the data reported for Human T-cell Lymphotropic Virus type-1 (HTLV-1), a genetically and functionally related retrovirus, where high PVLs in pregnant mothers are associated with a greater probability of transmitting the infection to children during delivery or by oral consumption of infected colostrum or milk [5]. Whether the infection is prenatal or postnatal, the rapid PVL rise suggests that the immune response does not stop viral replication. This is supported by data that show that while the calf immune system is functional at birth, it is immature until at least 4 months of age [6]. Calves are immunosuppressed during the first week of life because of estrogen and cortisol produced by the dam before parturition and cortisol produced by the calf during parturition [7]. Some cytokines that regulate BLV expression and delay progression to PL in adult cattle, including IFN-γ, IL-2, and IL-4 [8,9], are not efficiently expressed during the first week of life. Moreover, reduced levels of C3d (a subfragment of complement protein C3) and the presence of maternal antibodies in serum may result in the suppression of neonatal B lymphocytes, by binding to their ligands CD21 (activation) and CD32 (supression), respectively [10]. Therefore, despite evidence of adequate colostrum intake and absorption (Figures 1 and 2), the adaptive and innate immune responses were not effective to maintain a balance between the response and the viral infection cycle, and, as a consequence, did not reach an equilibrium point within 3 to 8 months, as recently reported for experimentally inoculated adult sheep and cattle [11,12]. This is critical in terms of viral transmission and infection epidemiology. We speculate that viral burst cycles occur during the first few months of life in perinatally infected calves, allowing rapid transmission by contact to susceptible neighbors. Similar observations have been made in HTLV-1 [13]. Moreover, patients infected with HTLV-1 as children are more prone to develop clinical signs [14]. While this was not detected in this study, a similar phenomenon could occur with BLV.

From an epidemiological point of view, the presence of animals with high PVLs is dangerous. Because it is a bloodborne pathogen, high levels of in vivo BLV infection are associated with higher probability of transmission [4]. Because BLV is highly endemic in dairy herds, classical control measures are not economically sustainable. Since BLV infection produces serious economic losses due to fatal lymphosarcoma and trade restrictions, alternative programs must be developed to reduce the spread of infection and gradually decrease infection prevalence. Elimination of adult cattle with high PVLs, along with movement of non-infected or low-infected heifers, may prove to be a feasible alternative, as previously discussed [1]. However, this approach requires uninfected heifers for movement. This study suggests that young infected animals represent a great risk for non-infected calves and could be a reason for the increased incidence before first parturition, when approximately half of pregnant heifers are already infected, 20–40% with high PVLs [15]. Propagation could occur through iatrogenic procedures or even by contact with secretions or excretions that could be infected with the virus. Moreover, virus could transfer the maternal barrier when these animals are pregnant spreading the infection to the offspring. A follow-up large-scale study with perinatally infected calves would be helpful to determine the prevalence of infection, range of PVLs, monitor transmission, whether these animals are more prone to develop lymphosarcoma, and to monitor offspring and the related incidence of infection.

## Conclusion

Calves infected during the first week of life could play an active role in early propagation of BLV to susceptible animals, since their PVL raised up during the first 12 months and persist as high for years. Early elimination could help to prevent transmission to young susceptible animals and to their own offspring. To our knowledge, this is the first study of the kinetics of BLV proviral load and antibody titers in newborn infected calves.

## Methods

### Animals

Study animals consisted of seven newborn calves with BLV detected by nested PCR during the first week of life. Six calves (#10902, #10934, #10938, #10960, #10964 and #10968) belonged to a typical dairy farm, with approximately 800 milking cows. Although this farm have used corrective management practices to prevent spread of BLV infection through iatrogenic procedures it had increasing BLV prevalence with age: 11.4% at birth, 15% at 15 months, 40% at 27% months, 61.7% after first delivery, and 86.5% in adult cows [16]. The remaining calf (#100) belonged to an experimental farm in our facilities (CNIA – INTA) with a BLV prevalence of 30%. Blood was taken by jugular venipuncture with heparin. Plasma and whole blood was stocked frozen until analyzed. Farm owners’ consent was obtained before animal sampling. The procedures followed for extraction and handling of samples were approved by the Institutional Committee for Care and Use of Experimental Animals of the National Institute of Agricultural Technology (CICUAE-INTA) under protocol number 35/2010 and followed the guidelines described in the institutional Manual.

### Antibody detection and titration

Anti-BLV antibodies were detected in plasma and colostrum using an ELISA test developed in-house, as previously reported [17]. Antibody titers were assayed by end-point dilution using two-fold dilutions of plasma. Titers were expressed as the reciprocal of the dilution.

### Proviral load detection and quantification

Total DNA was extracted from frozen whole blood or whole colostrum using the High Pure PCR Template Preparation kit (Roche, Penzberg, Germany) per the manufacturer’s instructions. BLV proviral DNA was detected by nested PCR [18] and quantified via real-time PCR [19] using FastStart Universal SYBR Green Master (ROX) (Roche). To determine proviral loads, a fragment of the *pol* gene [19] was amplified using 50 ng of DNA as a template. A standard curve was generated by amplification of serial 10-fold dilutions of total DNA extracted from fetal lamb kidney (FLK) cells containing four copies of BLV proviral DNA per cell (from 50 to 0.005 ng of total DNA corresponding to 85500 to 8.55 copies of the BLV genome, respectively). The constitutive *18S* ribosomal gene was amplified in parallel as a reference [19]. Due to the lack of an internationally harmonized threshold to differentiate between low and high proviral load we proposed and used DNA from FLK-BLV cells in a final concentration of 1% in non-infected PBMCs as a threshold. This cut-off point was stated using our own criteria and based on published data [20] on the number of infected cells in vivo in AL and PL animals, as PL animals have more infected lymphocytes circulating, and thus, higher proviral loads and higher probability of transmission of infection [4]. The internal FLK 1% calibrator corresponds to 0.04 BLV copies/cell in our system.

## Abbreviations

BLV: Bovine leukemia virus; PVL: Proviral load; PBMCs: Peripheral blood mononuclear cells; PL: Persistent lymphocytosis; AL: Aleukemic; Ab: Antibody; HTLV-1: Human T Lymphotropic Virus type-1; IFN-γ: Interferon-gamma; IL-2: Interleukin-2; IL-4: Interleukin-4; DNA: Deoxyribonucleic acid; PCR: Polymerase chain reaction; ng: Nanogram; FLK: Fetal lamb kidney.

## Competing interests

The authors declare that they have no competing interests.

## Authors’ contributions

GG carried out assays, analyzed data, and drafted the manuscript. IA helped with nested and real-time PCR. RM helped with ELISAs. FR provided the samples and assisted in study planning. KT planned the study, analyzed data, and drafted the manuscript. All authors read and approved the final manuscript.
